# Subjective satiety and other experiences of a Paleolithic diet compared to a diabetes diet in patients with type 2 diabetes

**DOI:** 10.1186/1475-2891-12-105

**Published:** 2013-07-29

**Authors:** Tommy Jönsson, Yvonne Granfeldt, Staffan Lindeberg, Ann-Christine Hallberg

**Affiliations:** 1 Center for Primary Health Care Research, Lund University/Region Skåne, Skåne University Hospital, Malmö, Sweden; 2 Department of Food Technology, Engineering and Nutrition, Lund University, Lund, Sweden

**Keywords:** Satiety, Diabetes, Weight loss, Paleolithic diet

## Abstract

**Background:**

We found marked improvement of glycemic control and several cardiovascular risk factors in patients with type 2 diabetes given advice to follow a Paleolithic diet, as compared to a diabetes diet. We now report findings on subjective ratings of satiety at meal times and participants’ other experiences of the two diets from the same study.

**Methods:**

In a randomized cross-over study, 13 patients with type 2 diabetes (3 women and 10 men), were instructed to eat a Paleolithic diet based on lean meat, fish, fruits, vegetables, root vegetables, eggs and nuts, and a diabetes diet designed in accordance with dietary guidelines, during two consecutive 3-month periods. In parallel with a four-day weighed food record, the participants recorded their subjective rating of satiety. Satiety quotients were calculated as the intra-meal quotient of change in satiety during a meal and consumed energy or weight of food and drink for that specific meal. All participants answered the same three open-ended questions in a survey following each diet: “What thoughts do you have about this diet?”, “Describe your positive and negative experiences with this diet” and “How do you think this diet has affected your health?”.

**Results:**

The participants were equally satiated on both diets. The Paleolithic diet resulted in greater satiety quotients for energy per meal (p = 0.004), energy density per meal (p = 0.01) and glycemic load per meal (p = 0.02). The distribution of positive and negative comments from the survey did not differ between the two diets, and the comments were mostly positive. Among comments relating to recurring topics, there was no difference in distribution between the two diets for comments relating to tastelessness, but there was a trend towards more comments on the Paleolithic diet being satiating and improving blood sugar values, and significantly more comments on weight loss and difficulty adhering to the Paleolithic diet.

**Conclusions:**

A Paleolithic diet is more satiating per calorie than a diabetes diet in patients with type 2 diabetes. The Paleolithic diet was seen as instrumental in weight loss, albeit it was difficult to adhere to.

**Trial registration:**

ClinicalTrials.gov: NCT00435240

## Background

Weight loss is often favourable in treatment of patients with type 2 diabetes and is facilitated by a satiating diet. We previously reported that a Paleolithic diet is more satiating per calorie than a Mediterranean-like diet [1,2]. We now report findings on subjective ratings of satiety from a randomized cross-over study on 13 patients with type 2 diabetes comparing a Paleolithic diet with a diabetes diet. We also report the participants’ answers to a survey taken after each diet comprising three open-ended questions on their experiences of the respective diet. From this study, we previously reported lower mean values of HbA1c, triacylglycerol, diastolic blood pressure, weight, BMI and waist circumference, and higher mean values of high-density lipoprotein cholesterol, after advice to follow a Paleolithic diet, as compared to a diabetes diet [3]. The Paleolithic diet was based on lean meat, fish, fruits, vegetables, root vegetables, eggs and nuts, while the diabetes diet aimed at evenly distributed meals with increased intake of vegetables, root vegetables, dietary fiber, whole-grain bread and other whole-grain cereal products, fruits and berries, decreased intake of total fat with more unsaturated fat, and replacement of high-fat dairy products with low-fat varieties. The main differences in food consumption, as reported in four-day weighed food records, were lower intakes of cereals and dairy products, and higher intakes of fruits, vegetables, meat and eggs, with the Paleolithic diet, as compared with the diabetes diet. Further, the Paleolithic diet had lower values for total energy, energy density, carbohydrates, dietary glycemic load, saturated fatty acids and calcium, and was richer in unsaturated fatty acids, dietary cholesterol and several vitamins. Dietary glycemic index (GI) was slightly lower in the Paleolithic diet (GI = 50) than in the diabetes diet (GI = 55) [3]. For background information on the concept of satiation and its determinants and the satiety quotient, see [1].

## Methods

### Patients

Approval of the study was obtained from the Regional Ethics Review Board in Lund, Sweden and the trial was registered at ClinicalTrials.gov (identifier: NCT00435240). The study was a randomized, cross-over, dietary intervention study in 13 patients with type 2 diabetes without insulin treatment (3 women and 10 men), recruited from three primary health care units in the Lund area in Sweden. We included adult patients with type 2 diabetes and a C-peptide value above zero, unaltered medical diabetes treatment and stable weight for three months before the start of the study, an HbA1c value above 5.5% (according to the Mono-S standard), a creatinine level below130 μmol/L, liver enzyme levels below four times their respective upper reference value, no chronic oral or injection steroid treatment and no acute coronary event or change in medication of beta blockers or thyroxin during the six months before the start of the study. Exclusion criteria during ongoing study were a change in beta blocker or thyroxine medication, chronic oral or injection steroid treatment, warfarin treatment, a creatinine level above 130 μmol/L or liver enzyme levels four times or more above their respective upper reference value, acute coronary events, and physical or psychological illness or changes in personal circumstances which would make further study participation impossible.

Recruitment for the study was performed during routine clinical work by diabetes nurses and medical doctors involved in the study, as previously described [3]. In addition, a letter containing written study information was sent by TJ to subjects at two of the health stations who, based on journal data, seemed to match the inclusion criteria. All recruited subjects were given oral and written study information prior to signing a consent form to participate in the study and were then further assessed for eligibility.

### Procedure

All eligible subjects were informed of the intention to compare two healthy diets in the treatment of type 2 diabetes and that it was unknown whether either of them would be superior to the other. At the study’s start, all eligible subjects were randomized to start with either a diabetes diet designed in accordance with current guidelines [4] or a Paleolithic diet. Randomization was performed by diabetes nurses by opening opaque, sealed envelopes (prepared by TJ) containing the name of the initial diet, with equal proportions of envelopes for both diets. After randomization, there was no blinding of dietary assignment to study participants, not to those administering the interventions or assessing the outcomes. Immediately after randomization, all subjects received oral and written information individually by diabetes nurses in the morning about their respective initial diet. After three months, all subjects switched diets and received new oral and written information individually by diabetes nurses about the diet for the following three months. Written information containing dietary advice and food recipes was similarly formulated for both diets. To increase conformity, the dietary advice and data collection procedures were discussed by all authors of the first report from this study (except YG) at several meetings prior to the start of the study [3]. Similar advice about regular physical activity was given to all subjects.

The information on the diabetes diet stated that it aimed to provide evenly distributed meals with increased intake of vegetables, root vegetables, dietary fiber, whole-grain bread and other whole-grain cereal products, fruits and berries, and decreased intake of total fat with more unsaturated fat. Most dietary energy should come from carbohydrates in foods naturally rich in carbohydrates and dietary fiber. The concepts of glycemic index and varying meals through meal planning by the Plate Model were explained [5]. Salt intake was recommended to be kept below 6 g per day.

The information on the Paleolithic diet stated that it should be based on lean meat, fish, fruit, leafy and cruciferous vegetables, root vegetables, eggs and nuts, while excluding dairy products, cereal grains, beans, refined fats, sugar, candy, soft drinks, beer and extra salt. The following items were recommended in limited amounts for the Paleolithic diet: eggs (≤2 per day), nuts (preferentially walnuts), dried fruit, potatoes (≤1 medium-sized per day), rapeseed or olive oil (≤1 tablespoon per day) and wine (≤1 glass per day). The recommended intake of the other foods was not restricted and no advice was given with regard to proportions of food categories (e.g. animal versus plant foods). The evolutionary rationale for a Paleolithic diet and potential benefits were explained [6].

### Evaluation

As previously reported, a 4-day weighed food record covering four consecutive days, including one weekend day, was completed by the participants, starting 6 weeks after they initiated each diet [3]. In parallel with keeping this food record, the participants recorded the time of day for each meal, including snacks, as well as their subjective rating of satiation at meal initiation and 30 minutes after meal initiation on a 7-point equal interval, bipolar scale of hunger/fullness, modified from Holt et al. 1992, as previously described [1]. This scale was anchored at −3 (“Very hungry”) with a midpoint of 0 (“No particular feeling”) and a highest score of + 3 (“Very full”). The participants were invited to place marks between the 7 points. The scale yields numeric results in units termed Rating Scale units (RS). Change in satiety during the meal was calculated as change in satiety between meal initiation and 30 minutes after meal initiation. Satiety quotients were calculated as the intra-meal quotient of change in satiety during the meal and consumed energy or weight of food and drink for that specific meal or calculated energy density, glycemic load or GI for that meal.

Following each 3-month period on a particular diet, all participants were asked to answer the same three open-ended questions in a survey on their experiences of the diet they had just eaten: “What thoughts do you have about this diet?”, “Describe your positive and negative experiences with this diet” and “How do you think this diet has affected your health?” The survey was taken in Swedish since all participants were Swedish. The answers and the three open-ended questions were then translated into English by TJ and Science Editor Stephen Gilliver, the latter a native English speaker.

### Statistics

Two-sided t-tests for dependent samples were used to test treatment effects when data were normally distributed (as determined by the Shapiro-Wilk test). Otherwise, the related-samples Wilcoxon signed-rank test was used. Two-sided t-tests for independent samples were used to compare mean values of outcome variables for the group starting with the Paleolithic diet compared with the group starting with the diabetes diet to check for carry-over effects [7]. Two-sided t-tests for independent samples were used to compare differences between the effects of the first and second diets on outcome variables for the group starting with the Paleolithic diet compared with the group starting with the diabetes diet to check for period effects [7]. We found no carry-over or period effects. P < 0.05 was considered to indicate statistical significance. Data and results are expressed as the mean ± standard deviation. Bivariate correlation and linear regression were used for post hoc analysis.

## Results

There was no significant difference between the diets in measures of subjective satiety at meal initiation and 30 minutes after meal initiation, or in change in satiety during meal (Table 1). There was also no difference between the diets in length of time between meals or number of meals per day (Table 1). The Paleolithic diet resulted in greater satiety quotients for energy per meal (1.8 ± 0.7 RS*MJ^-1^ vs. 1.5 ± 0.5 RS*MJ^-1^, Paleolithic vs. diabetes, p = 0.004, Table 1), energy density per meal (0.5 ± 0.2 RS*g*kJ^-1^ vs. 0.4 ± 0.1 RS*g*kJ^-1^, Paleolithic vs. diabetes, p = 0.01, Table 1) and glycemic load per meal (297 ± 138 RS*kg^-1^ vs. 153 ± 170 RS*kg^-1^, Paleolithic vs. diabetes, p = 0.02, Table 1). There were no differences between the diets in satiety quotients for weight per meal and GI per meal (Table 1).

**Table 1:** Effect of Paleolithic diet compared to diabetes diet on satiety

	**Paleolithic diet**	**Diabetes diet**	P*	P**
Time between meals (hours:minutes)	03:12 ± 00:39	03:09 ± 00:33		0.8
Meals per day (n)	4.9 ± 1.0	5.0 ± 1.0	0.7	
Satiety at meal initiation (RS)	−1.0 ± 0.4	−1.0 ± 0.4	0.5	
Satiety 30 minutes after meal initiation (RS)	1.2 ± 0.5	1.2 ± 0.4	0.6	
Change in satiety 30 minutes after meal initiation (RS)	2.2 ± 0.7	2.2 ± 0.7	1.0	
Satiety quotient for weight per meal (RS/kg)	8.7 ± 3.8	9.8 ± 5.0	0.2	
Satiety quotient for energy per meal (RS/MJ)	1.8 ± 0.7	1.5 ± 0.5	0.004	
Satiety quotient for energy density per meal (RS*g/kJ)	0.5 ± 0.2	0.4 ± 0.1	0.01	
Satiety quotient for glycemic load per meal (RS/kg)	297 ± 138	153 ± 170		0.02
Satiety quotient for glycemic index per meal (RS)	0.043 ±0.014	0.040 ± 0.013	0.3	

Effect of 12 weeks of Paleolithic diet compared to 12 weeks of diabetes diet on measures of satiety in a randomized cross-over study on 13 patients with type 2 diabetes (group mean ± SD). Estimated from 4 day weighed food records with rating scale used to assess subjective satiety in Rating Scale units (RS). Two-sided t-tests for dependent samples (P*) were used to test treatment effects when data were normally distributed (as determined by the Shapiro-Wilk test). Otherwise, the related-samples Wilcoxon signed-rank test was used (P**).

In post hoc analysis of within-subject differences (value during Paleolithic diet minus value during diabetes diet), satiety quotients for energy per meal correlated with triglyceride levels and vitamin B6 intake (Pearson’s correlation 0.60 and 0.64, p = 0.03 and 0.02, respectively, Table 2), satiety quotients for energy density per meal correlated with water from food (Pearson’s correlation 0.71, p = 0.01, Table 2), and satiety quotients for glycemic load per meal correlated with BMI and spirits (Pearson’s correlation −0.84 and 0.59, p = 0.0003 and 0.03, respectively, Table 2).

**Table 2:** Exploratory analyses performed on satiety, outcome and dietary variables

	Bivariate correlation P*	**Pearson correlation r**	**Linear regression P**^**§**^	**Adjusted R**^**2**^
∆SQ for energy per meal versus outcome variables				
∆ TG (mmol/l)	0.03	0.60	0.03	0.30
∆SQ for energy per meal versus dietary variables				
∆Vitamin B6 (mg)	0.02	0.64	0.02	0.35
∆Potassium (mg)	0.03	0.60	NS	
∆Total energy (MJ)	0.049	0.55	NS	
∆SQ for energy density per meal versus outcome variables				
No correlations				
∆SQ for energy density per meal versus dietary variables				
∆Water from food (g)	0.01	0.71	0.01	0.45
∆Phosphorus (mg)	0.01	0.67	NS	
∆Potassium (mg)	0.01	0.66	NS	
∆Vitamin B6 (mg)	0.02	0.64	NS	
∆Calcium (mg)	0.03	0.59	NS	
∆Fiber (g)	0.04	0.58	NS	
∆Total energy (MJ)	0.046	0.56	NS	
∆Carbohydrate (g)	0.047	0.56	NS	
∆SQ for glycemic load per meal versus outcome variables				
∆BMI (kg/m^2^)	0.0003	−0.84	0.0003	0.69
∆Weight (kg)	0.03	−0.59	NE	
∆Waist (cm)	0.02	−0.63	NS	
∆SQ for glycemic load per meal versus dietary variables				
∆Spirits (g)	0.03	0.59	0.03	0.29

Exploratory analyses performed on satiety, outcome and dietary variables with significant effects from a Paleolithic diet as compared to a diabetes diet plus water from food and energy-containing beverages (spirits, wine, beer, sweet beverages and juice). Analyses consisted of bivariate correlations between within-subject differences (∆, value during Paleolithic diet minus value during diabetes diet) in satiety quotients (SQ) for energy, energy density and glycemic load per meal versus within-subject differences in outcome and dietary variables. Significantly correlating variables were entered into a stepwise forward linear regression analyses. *P for bivariate correlation between variables in a two-sided *t*-test. §Stepwise forward linear regression analyses entering significantly correlated variables. *NS* Not Significant, *NE* Weight and BMI not entered in same regression analysis due to calculatory relatedness.

All participants answered the survey after each diet. TJ and ACH analyzed all comments for recurrent types of answers. Seven common types of answers were recognized and their frequencies determined (positive, negative, tasteless, improved blood sugar, satiating, lose body weight and difficult adhering to, Additional file 1). The distribution of positive and negative comments did not differ between the two diets and the comments were mostly positive for both diets (Table 3). There was no difference in distribution between the two diets in terms of tastelessness. However, there were trends towards more comments on the Paleolithic diet improving blood sugar values (3 vs. 0, Paleolithic vs. diabetes, p = 0.08, Table 3) and being satiating (5 vs. 0, Paleolithic vs. diabetes, p = 0.06, Table 3), and significantly more comments on weight loss (10 vs. 2, Paleolithic vs. diabetes, p = 0.02, Table 3) and difficulty adhering to the diet (13 vs. 4, Paleolithic vs. diabetes, p = 0.02, Table 3).

**Table 3:** Participants’ comments on the two diets

	**Diabetes diet**	**Paleolithic diet**	P*
Positive	17	16	0.8
Negative	4	3	0.7
P**	0.03	0.01	
Tasteless	1	4	0.2
Improved blood sugar	0	3	0.08
Satiating	0	5	0.06
Lose body weight	2	10	0.02
Difficulty adhering to	4	13	0.02

All participants answered the same three open-ended questions in a survey following each diet: “What thoughts do you have about this diet?”, “Describe your positive and negative experiences with this diet” and “How do you think this diet has affected your health?” Seven common types of answers were recognized and their frequencies determined; positive, negative, tasteless, improved blood sugar, satiating, lose body weight and difficult adhering to. P* related-samples Wilcoxon signed rank test on number of comments on each diet. P** related-samples Wilcoxon signed rank test on number of positive and negative comments on diabetes diet and Paleolithic diet, respectively.

## Discussion

### Key findings

There was no difference in subjectively assessed satiation between the diets. The Paleolithic diet resulted in greater satiety quotients for energy per meal, energy density per meal and glycemic load per meal. The Paleolithic diet was thus more satiating per calorie compared to the diabetes diet, which corroborates our previous study showing that the Paleolithic diet was more satiating per calorie than a Mediterranean diet [1]. The distribution of positive and negative comments from the survey did not differ between the two diets and the comments were mostly positive for both diets. Among comments relating to recurring topics, there was no difference in distribution between the two diets in terms of tastelessness, but there was a trend towards more comments on the Paleolithic diet being satiating and improving blood sugar values, and significantly more comments on weight loss and difficulty adhering to the Paleolithic diet.

### Possible mechanisms and explanations

Both diets were equally satiating, as measured by subjective satiety recorded in parallel with a 4-day weighed food record. Mean satiety for both diets 30 minutes after meal initiation was 1.2 Rating Scale units, which is between “somewhat satisfied” and “satisfied” and is slightly lower than previous results [1]. This probably still indicates adequate satiety, since the ratings are from all meals including snacks, with an average of about five meals per day on both diets, slightly more than in previous studies [1]. In support of this conclusion, five comments from the survey, all relating to the Paleolithic diet, were on being satiated (e.g. “no difficulty becoming full” and “have not been overly hungry at any time”) and only one participant, when on the Paleolithic diet, commented on being hungry (“almost always hungry”).

The Paleolithic diet was more satiating per calorie, despite its lower content of supposedly satiating fiber [8], which also didn’t correlate with the satiety quotient for energy. This greater satiating capacity may instead have been caused by the lower energy density of the paleolithic diet, as evidenced by the significantly higher satiety quotient for energy density for the Paleolithic diet [9,10]. Water incorporated into a food increases its satiating capacity by reducing its energy density [11], and although we found no difference between the diets in calculated water content, there was a significant correlation between water content and satiety quotient for energy density. Differences in beverage intake could also potentially have affected satiety [12], but we found no such differences between the diets, although there was a correlation between spirits and satiety quotient for glycemic load.

Consistent with our previous results [1], there was a higher relative intake of protein with the Paleolithic diet (24 ± 3% of dietary energy) compared to the diabetes diet (20 ± 4%9. High-protein diets reportedly reduce appetite and ad libitum caloric intake [13-15]. However, as in our previous study, there was no correlation between relative protein intake and satiety quotients, and no difference in absolute intake of protein between the two diets, making the relative difference in protein intake an unlikely explanation for the difference in satiety.

Just as in our previous study, we found significantly lower carbohydrate intakes in both absolute and relative terms with the Paleolithic diet, a candidate explanation for our observed effect on satiety per calorie [16], but there was no correlation between satiety quotients and carbohydrate intake. Intakes of sources of carbohydrates with previously reported differences in effects on satiety, such as cereals and fruits, differed between the diets [9], but also didn’t correlate with differences in satiety quotients. Unlike our previous study [1], there was no difference in sodium intake between the diets [3], possibly also indicating no difference in salt intake. Assuming a 1:2.5 weight relationship between intake of sodium and salt (NaCl), the participants’ estimated average daily consumption of salt was 6.3 g on the Paleolithic diet compared to 7.4 g on the diabetes diet. There were some comments on the Paleolithic diet concerning salt (e.g. “miss salt”, “Tasteless without salt” and “You miss salt”), which could indicate less added salt and lower palatability, with effects on satiety [17]. However, since we didn’t measure sodium excretion in urine, a better measure of salt intake, we don’t know whether there was any difference between the diets in terms of salt intake. As mentioned above, the intake of sodium didn’t differ between the diets, nor did comments on palatability. Differences between diets in the intake of vitamin B-6 were correlated with differences in satiety quotients for energy per meal, although the clinical significance of this is unclear.

Among the various comments, there was a trend for noticing improved glucose homeostasis on the Paleolithic diet (e.g. “Blood sugar has stabilized.”, “HbA1c decreased from 6.2 to 5.7”), and significantly more comments on losing weight (e.g. “Only noticed weight loss”, “I have lost weight”, “You lose weight”). The comments probably reflect the participants’ awareness during the study of dietary effects, which they can both notice directly and perceive as important. The comments also reflected differences between the diets in weight loss and glucose homeostasis [3]. There were more comments on the Paleolithic diet being difficult to adhere to (e.g. “The variety has meant that my wallet has taken a hit.”, “I miss bread a lot”, “Somewhat monotonous diet” and “Difficult to adhere to. Miss milk and cereal products”) compared to the diabetes diet (e.g. “can’t do anything spontaneously, must plan”, “However, it can be difficult to get invited out when you can’t just eat anything.”). This is not very surprising, since the Paleolithic diet necessitates greater changes to most people’s diet compared to the diabetes diet. Importantly, the comments on the Paleolithic diet were not overly negative and some were also positive (e.g. “I strongly believe in it and will certainly use much of it in the future.”).

### Comparison with findings from other studies

This is the first study to report participants’ experiences of a Paleolithic diet and the second study to report on subjective satiety from a Paleolithic diet [1]. Our findings corroborate and emphasize previous findings that the Paleolithic diet is more satiating per calorie than other diets (in this study a diabetes diet).

### Limitations of the present study

Since we didn’t measure urine excretion of sodium, we can’t be sure of salt intake. Future studies should if possible include urine measurements of sodium and calcium.

### Clinical and research implications

Our findings corroborate previous findings that the Paleolithic diet is more satiating per calorie than other diets. This is an important aspect which could make the Paleolithic diet ideally suited to prevent and treat obesity and associated diseases.

## Conclusions

A Paleolithic diet is more satiating per calorie than a diabetes diet in patients with type 2 diabetes. This corroborates our previous findings and is important in facilitating the often desirable weight loss in the treatment of patients with type 2 diabetes. The participants perceived the Paleolithic diet to be instrumental in their losing weight, albeit it was difficult to adhere to.

## Availability of supporting data

The data set supporting the results of this article is included within the article (and its additional file).

## Competing interests

The authors declare that they have no competing interests.

## Authors’ contributions

TJ participated in the design and execution of the study, participated in statistical analysis, and conceived and wrote the article. ACH participated in analysis of the survey and the design of the article, as well as revising it for important intellectual content. YG participated in the design of the article as well as revising it for important intellectual content. SL participated in the design of the study and the design of the article, as well as revising it for important intellectual content. All authors read and approved the final manuscript.

## Supplementary Material

Additional file 1 **Supporting data set.** Data set supporting our results on satiety and post hoc analysis (worksheet ‘Data set’) plus survey answers in both original Swedish form (worksheet ‘Survey in Swedish’) and translated into English (worksheet ‘Survey in English’). Each row numbered 1–13 in first column ‘Person-ID’ corresponds to data and survey answers from one participant. Each column heading contains a variable name stating what individual mean has been measured, in what unit and whether on the Paleolithic diet, diabetes diet or if it is the difference between the two diets (DeltaPd, value during Paleolithic diet minus value during diabetes diet). The variable names and their description are listed below. Click here for file
